# The political economy of priority-setting for health in South Sudan: a case study of the health pooled fund

**DOI:** 10.1186/s12939-022-01665-w

**Published:** 2022-05-16

**Authors:** Heloise Widdig, Noor Tromp, George William Lutwama, Eelco Jacobs

**Affiliations:** 1 KIT Royal Tropical Institute, Amsterdam, The Netherlands; 2 Health Pooled Fund, Juba, South Sudan

**Keywords:** Priority-setting, Political economy analysis, Fragile and conflict affected settings, Basic packages of health services, Health financing

## Abstract

**Background:**

In fragile and conflict affected settings (FCAS) such as South Sudan, where health needs are immense, resources are scarce, health infrastructure is rudimentary or damaged, and government stewardship is weak, adequate health intervention priority-setting is especially important. There is a scarcity of research examining priority-setting in FCAS and the related political economy. Yet, capturing these dynamics is important to develop context-specific guidance for priority-setting. The objective of this study is to analyze the priority-setting practices in the Health Pooled Fund (HPF), a multi-donor fund that supports service delivery in South Sudan, using a political economy perspective.

**Methods:**

A multi-method study was conducted combining document review, 30 stakeholder interviews, and an examination of service delivery. An adapted version of the Walt and Gilson policy analysis triangle guided the study’s design and analysis.

**Results:**

Priority-setting in HPF occurs in a context of immense fragility where health needs are vast, service delivery remains weak, and external funding is essential. HPF's service package gives priority to the health of mothers and children, gender-sensitive programming, immunization services, and a community health initiative. HPF is structured by a web of actors at national and local levels with pronounced power asymmetries and differing vested interests and ideas about HPF’s role. Priority-setting takes place throughout program design, implementing partner (IP) contract negotiation, and implementation of the service package. In practice the BPHNS does not provide adequate guidance for priority-setting because it is too expansive and unrealistic given financial and health system constraints. At the local level, IPs must manage the competing interests of the HPF program and local health authorities as well as challenging contextual factors, including conflict and shortages of qualified health workers, which affect service provision. The resulting priority-setting process remains implicit, scarcely documented, and primarily driven by donors’ interests.

**Conclusion:**

This study highlights power asymmetries between donors and national health authorities within a FCAS context, which drive a priority-setting process that is dominated by donor agendas and leave little room for government ownership. These findings emphasize the importance of paying attention to the influence of stakeholders and their interests on the priority-setting process in FCAS.

## Background

Priority-setting is an intractable challenge faced by health planners worldwide because demand for healthcare inevitably exceeds the resources available to finance healthcare. In its most basic form, priority-setting is the process of making decisions about how best to allocate scarce resources to improve population health [1]. This challenge is especially difficult in low and middle income countries (LIMCs) and fragile and conflict affected settings (FCAS) where health needs are immense, financial resources are scarce, health infrastructure is rudimentary or damaged, and government stewardship is limited [2]. In these settings there is an absence of systematic processes to guide decision-making, a lack of reliable information to inform decisions, and a presence of multiple actors with differing agendas [3]. As a result, priority-setting tends to be ad hoc, rather implicit, and materializes through a haphazard series of opaque choices, which reflect competing interests of governments, donors, and other stakeholders [4, 5]. Despite the political nature of priority-setting, limited attention has been paid to the processes, interests, and institutions that shape priorities, characterize decisions on budgets, coverage of services, and interventions in LICs and especially in FCAS. Understanding these forces is essential to understanding the full priority-setting landscape within which priorities are determined.

Until now, the majority of priority-setting literature has assessed priority-setting processes against universalist ethical frameworks, most notably, the accountability for reasonableness framework [6–9]. While these frameworks recognize the essentiality of focusing on the priority-setting process, there is less focus on the underlying politics and interest. This introduces a risk of overlooking the complexity of the dynamics influencing priority-setting, leading to recommendations that are ill-suited to the political context, particularly in areas of fragile statehood. A political economy analysis (PEA) mitigates this risk as it focuses on the political, institutional, and context constraints under which priorities are set [10, 11]. In turn this context shapes underlying interests, incentives, institutions and how they interact in explicit and implicit priority-setting practices. It is essential to explore and understand these as they help to explain why priorities are set the way they are. In an effort to address the scarcity of the research examining the political economy of priority-setting in the context of fragile and conflict affected settings, this study uses a political economy framework to capture and describe the practices and experiences that define the priority-setting landscape in the context of South Sudan through the examination of the Health Pooled Fund (HPF), a pooled fund supporting the delivery of healthcare in South Sudan.

South Sudan is one of the world’s most fragile settings, ranking 3rd on the 2020 fragile states index [12]. The aftermath of its colonial history is characterized by one of the longest violent conflicts in Africa. Since its independence in 2011, South Sudan remains in a serious humanitarian crisis. The sustained conflict has greatly impaired the health system and contributed to an immense and diverse disease burden. The life expectancy in South Sudan is 58. With a maternal mortality ratio at 789 per 100,000 live births and infant mortality ratio of 71 deaths per 1000 live births, South Sudan ranks among the highest in the world for these measures [13].

As a result of the immense health needs and instability in the health system, South Sudan is highly dependent on donor contributions. One such funding mechanism is HPF, which is at the center of this study. HPF is a multi-donor fund that supports delivery of the national Basic Package of Health and Nutrition Services (BPHNS) in 80% of health facilities in eight out of the ten states in South Sudan. 1 HPF became active in South Sudan in 2012, and is in the third phase (HPF3) as of 2018. HPF is managed by a consortium of organizations which contracts non-governmental organizations (NGOs) as implementing partners (IPs) for particular geographical areas, named ‘lots’ to directly support primary health care delivery in selected health facilities in each lot. Within the HPF program, priority-setting of activities takes place but this has not yet been analyzed and documented.

## Methods

### Study design and conceptual framework

**Fig. 1:**
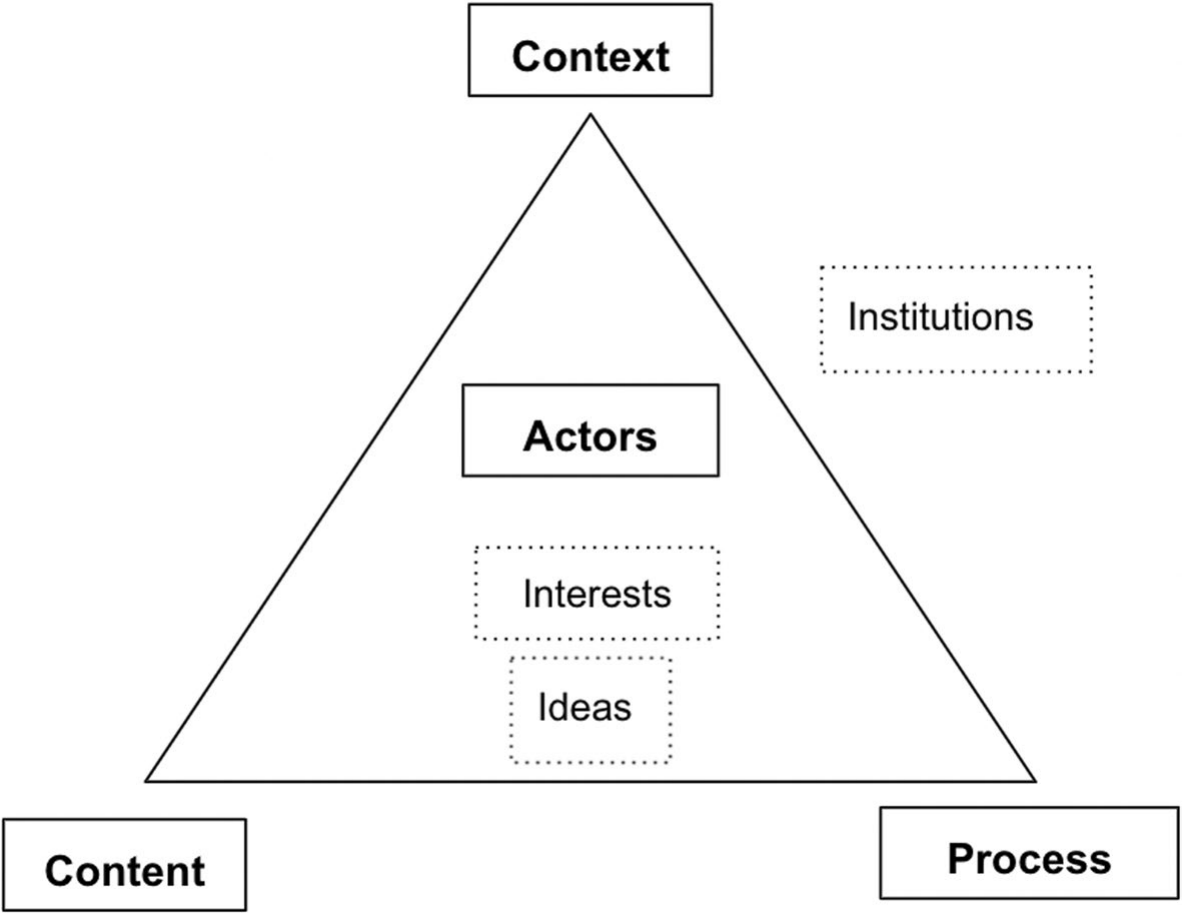
Adapted Walt and Gilson policy triangle conceptual framework for PEA

The study is structured around a political economy framework, developed to examine important elements of the priority-setting landscape of HPF. This framework guided data collection and analysis. A retrospective case study approach was chosen given its usefulness for examining complex social processes [14]. A multi-method, critical realist approach was used, combining key informant interviews, document review and quantitative examination of service delivery at the facility level [15]. An adapted version of the Walt and Gilson (1994) policy analysis triangle was applied as a conceptual framework (Fig. 1) [16], which examines the context, process, content, and actors that interact to shape a policy environment. This framework is grounded in the political economy perspective, and considers how these elements interact to shape policy-making. The framework examines the context (why), content (what), process (how) and actors (who) that interact to shape a policy environment [17]. We added the concepts of *institutions*, *interests*, and *ideas*, which according to Smith et al. 2014, helps to better uncover the political economy of the policy environment by explicitly emphasizing the interests of multiple stakeholder groups that interact and compete within the context of entrenched institutions and ideas [18]. For the sake of organizing the information, these concepts are presented as distinct concepts, however, they are inherently intertwined and it is these interactions that shape the priority-setting landscape.

### Operationalization of the conceptual framework

For context*,* we examined the history and the development of the health sector in the post conflict era (from 2011 to today) and the current health needs of the population in South Sudan that influence the HPF program and priorities set. For the *content,* we studied the outcomes of the priority-setting processes, including the focus of the HPF3 program as compared to HPF1 and HPF2, and what services are delivered at facility level by IPs within HPF3. For *actors*, we mapped the key stakeholders involved in HPF3, their roles and the power dynamics between them. *Power* is conceptualized in dispositional (money, knowledge, reputation), relational (A influencing B) and organizational terms (organizations, rules, bargaining) [19]. Furthermore, we explored their interests (desire or motivation to accomplish a certain objective [10]) concerning HPF programming, ideas (the way problems are framed [18]), and how these influence the priority-setting process. *Institutions* are defined as the sets of rules that guide and constrain behavior of individual actors [19]. We studied both the formal institutions (governing structures, partnership agreements, and accountability mechanisms) [18] and the informal institutions (rules not formally articulated or specified) that shape the rules of engagement in the priority-setting processes [19]. For the *process,* we studied the priority-setting process throughout the design of the HPF3 program at the national level and subsequently its implementation by IPs at the local level.

### Key informant interviews

Between April and June 2020 we conducted 30 interviews with key informants involved in HPF3. Purposive sampling was used to select key informants from each stakeholder group at the national, state, and district levels based on their involvement in HPF. This included key personnel from HPF donors (*n* = 4), the National Ministry of Health (MoH) of South Sudan (*n* = 2), the State MoH (SMoH) (*n* = 3), County Health Departments (CHD) (*n* = 2), the HPF management team (*n* = 5) and IP management (*n* = 14). The breakdown of key informants interviewed is provided in Table 1.

**Table 1:** Key informant characteristics

Institution	Male	Female	Total	Code
Donors	1	3	4	Donor_#
National MoH	2	-	2	MOH_#
SMoH	3	-	3	SMOH_#
CHD	2	-	2	CHD_#
HPF Secretariat	3	2	5	HPF_#
IP Management	11	3	14	IP_#
Total	22	8	30	

A semi structured interview guide was used containing topics related to the adapted Walt and Gilson conceptual framework. Interviews focused on HPF3, although necessary references to HPF1 and HPF2 were also made. All interviews were conducted online in English and lasted for one hour. All interviewees gave informed consent. All interviews were recorded and all but four were transcribed. For these four interviews (one CHD and three SMoH) only notes were taken as the audio recording quality did not allow for transcription.

### Document review

To contextualize the data collected during the interviews several documents related to health policy and HPF in South Sudan were reviewed. The selection of these documents was purposeful and guided by key informants, who recommended, referred to, or cited these documents. The BPHNS was reviewed to understand the contents of the national package. The Health Sector Development Plan (HSDP) (2012–2016) was reviewed to understand the development of the health sector and its structure. The HPF3 Business Case informed our understanding of the strategic direction of HPF3. Lastly, the HPF3 Request for Proposals (RFP) was reviewed to understand the service package IPs were expected to provide in facilities.

### Examination of service delivery

To understand service prioritization by IPs in service delivery and to provide further context when engaging with IPs, the proportion of primary health care centers (PHCCs) offering a selection of key health services were compared across lots. The focus was placed on PHCCs because they are the most comprehensive provider of primary care services across all lots. Data was retrieved from HPF’s 2019 facility service register, which includes self-reported lot level information on the availability of a full range of services and equipment at each facility. In the dataset, 1 indicates that the service(s) are available and 0 indicates that the facility does not provide the service. For each service there is a definition of what availability of the service means in practice. This information is reported quarterly by each IP using a District Health Information Software as part of HPF’s regular monitoring and evaluation processes. This data is not publicly available. We analyzed the availability of several services that compose HPF’s main activities: family planning, maternal and neonatal health, routine vaccination, and Gender Equality and Social Inclusion (GESI) services, mental health and psychosocial support (MHPSS) and sexual and gender based violence (SGBV) and disability services at PHCCs.

### Data Analysis

Using Atlas.ti (version 8), interview transcripts were coded using deductive thematic analysis guided by the conceptual framework. As topics emerged within each theme during the coding process, these were added as codes during the coding process. For each code the data was summarized to identify the main findings. To guide the reporting and analysis of the main findings for each theme, framing questions were used. These are presented in Table 2.

**Table 2:** Key themes, topics, and framing questions to guide data reporting and analysis

Theme	Topic	Framing Questions
Context	Historical legacies	What is the past history of the sector, including previous health sector reforms and experience of crisis? How has this impacted the health sector and health needs?
Content	HPF programming	What does HPF programming consist of? What relevant policy frameworks inform programming?
	Shifts in HPF programming and HPF3 programming	What shifts have occurred over the roll-out of HPF? What key shifts have determined HPF3 programming?
Institutions	Formal Institutions	Which governing structures and formal mandates inform the priority-setting environment?
	Informal Institutions	Which informal institutions and mandates inform the priority-setting environment?
	Rules of engagement	Which rules of engagement dictate stakeholders’ involvement in priority-setting?
Actors	Roles	Who are the key stakeholders in HPF? What are their roles?
	Interests + Ideas	What are the key interests of each stakeholder with regards to HPF programming? What ideas inform these interests? How do these interests play out in practice?
Process	Chronology	What are the steps of the priority-setting process(es)
	Explicit elements of the process	Which explicit priority-setting processes inform HPF service coverage, programming, and implementation?
	Implicit elements of the process	Which implicit priority-setting processes inform HPF service coverage, programming, and implementation?

To examine service provision, we looked at the percentage of PHCCs providing each service. This provided us with a picture for each lot, of the availability of the services that make up HPF’s stated service package. We also looked at the variation of the service delivery across lots to see if there were any large differences. This information importantly provided lot-level context for the interviews and a basis to investigate the ways in which constraints to service provision were successfully and unsuccessfully managed.

### Ethics

A waiver for ethical approval for this study was provided by the Royal Tropical Institute Research Ethics Committee (S-120). All research procedures were in accordance with the Declaration of Helsinki.

## Results

### Context in which HPF priorities are set

After the signing of the Comprehensive Peace Agreement in 2005, the Southern Sudan government and the development community gave priority to building a functioning health system [19]. Essential governing institutions were established at national and state levels and a policy framework was developed with extensive involvement of external actors. Post-independence, the newly established Government of the Republic of South Sudan (GRSS) renewed its commitments to universal health coverage and continued the development of its health policy frameworks [20]. Health policy frameworks such the HSDP (2012–2016), National Health Policy (2015–2024), and a revised BPHNS (2011) were developed with extensive involvement of external actors [20, 21].

Due to the sustained conflict, health needs remain vast. In addition to high maternal mortality, communicable diseases are a leading cause of morbidity and mortality, including lower respiratory infections, diarrheal disease, and tuberculosis [22]. Neglected tropical diseases are endemic and HIV affects an estimated 3% of the population. Though not quantified, non-communicable diseases also contribute to the disease burden, including cardiovascular disorders, diabetes, mental health disorders, violence related trauma, and substance abuse. Estimates indicate that 83% of the population lives in rural areas and 44% live within five kilometers of a functional health facility, posing a considerable barrier to service delivery) [21].

The protracted crisis has also significantly impacted the health system and service delivery remains weak. There is a significant qualified staff shortage, inadequate facilities, limited management capacity, high turnover of health authorities, and weak accountability systems. The estimated density of doctors and nurses is 0.15 and 0.25 per 10,000 population, respectively [20]. Furthermore, on-going clashes due to political conflict and local community rivalries affect the accessibility of health facilities.

The health sector heavily relies on external support. Government expenditure is 11% of the total health expenditure, while development and humanitarian partners finance 62%. Out-of-pocket health expenditure fills the remaining gap (22%) [23]. Besides HPF there are several other health and nutrition funding mechanisms. The World Bank funds the Provision of Essential Services Project, which supports health facilities in the two States not supported by HPF (Upper Nile and Jonglei). Furthermore, several vertical programs operate through bilateral agreements with the MoH, including programs for HIV, malaria, and tuberculosis by the Global Fund, a nutrition program funded by UNICEF, supplementary feeding commodities by the World Food Program, and a program for family planning and reproductive health commodities by the UNFPA. While the HSDP states that coordination is important for aid effectiveness [20], communication and integration remains a constant challenge. HPF is perceived as a main contributor in terms of service delivery as was reiterated by respondents.

*...support is solely offered by HPF… so there is nothing that the MoH chips in terms of support for the health facilities, so basically HPF is running those health facilities. –* HPF_01

### Content: the priorities set at national and local level

HPF3’s stated aim is to support the GRSS in delivering the BPHNS through the provision of services through a network of health facilities and community-based systems. Although elements of the HPF program have remained consistent, changes have occurred over the course of roll-out, reflecting a changing context, priorities, and interests. Informants revealed general shifts between the first to third phase and specific shifts in the creation of HPF3 programming. While HPF1 focused on post-independence health systems *building*, HPF2 focused on *strengthening* foundational structures from HPF1 and *maintaining* service provision facilities while in the midst of renewed conflict. In HPF3, the aim shifted to *health system stabilization*, with emphasis on local structures and community ownership. While HPF2 supported all facilities across the eight states to increase access to services, HPF3 reduced this number and halted support for facilities that were (partially) destroyed, located in deserted areas, offered a negligible number of health services in practice, or were close to similar or better functioning facilities to concentrate funding, decrease waste and improve quality of care at functioning facilities in populated areas. Another shift occurred towards an incentive payment system. Prior to HPF3, IPs paid health staff salaries, which created challenges as organizations paid different rates. HPF decided to use the harmonized incentive scale established by MoH to balance payments across counties. With this incentive scale in place, HPF switched away from paying salaries to paying incentives at rates stipulated by the government. This shifted the responsibility of paying health worker salaries to the MoH.

Within HPF3, explicit priority is given to the health of mothers, pregnant women and children under five, which covers only a portion of the BPHNS. Furthermore, the HPF3 service package emphasized and allocated funding to three significant components: 1) the Boma Health Initiative (BHI) supporting community health workers and community engagement, 2) GESI services supporting gender-based violence, clinical management of rape, family planning, and disability and mental health services and 3) immunization services. These service areas had not previously been key components in the HPF2 service package. For example the emphasis on GESI services was new in HPF3.

*A new concept came up...from the donors where they want us to mainstream mental health and disability programming… This year there is funding that can be slotted in for GESI issues...when the EU came into the pool of donors. [The] additional funding...allowed them to reprogram some of the money and utilize it for these activities. –* HPF_01

**Fig. 2:**
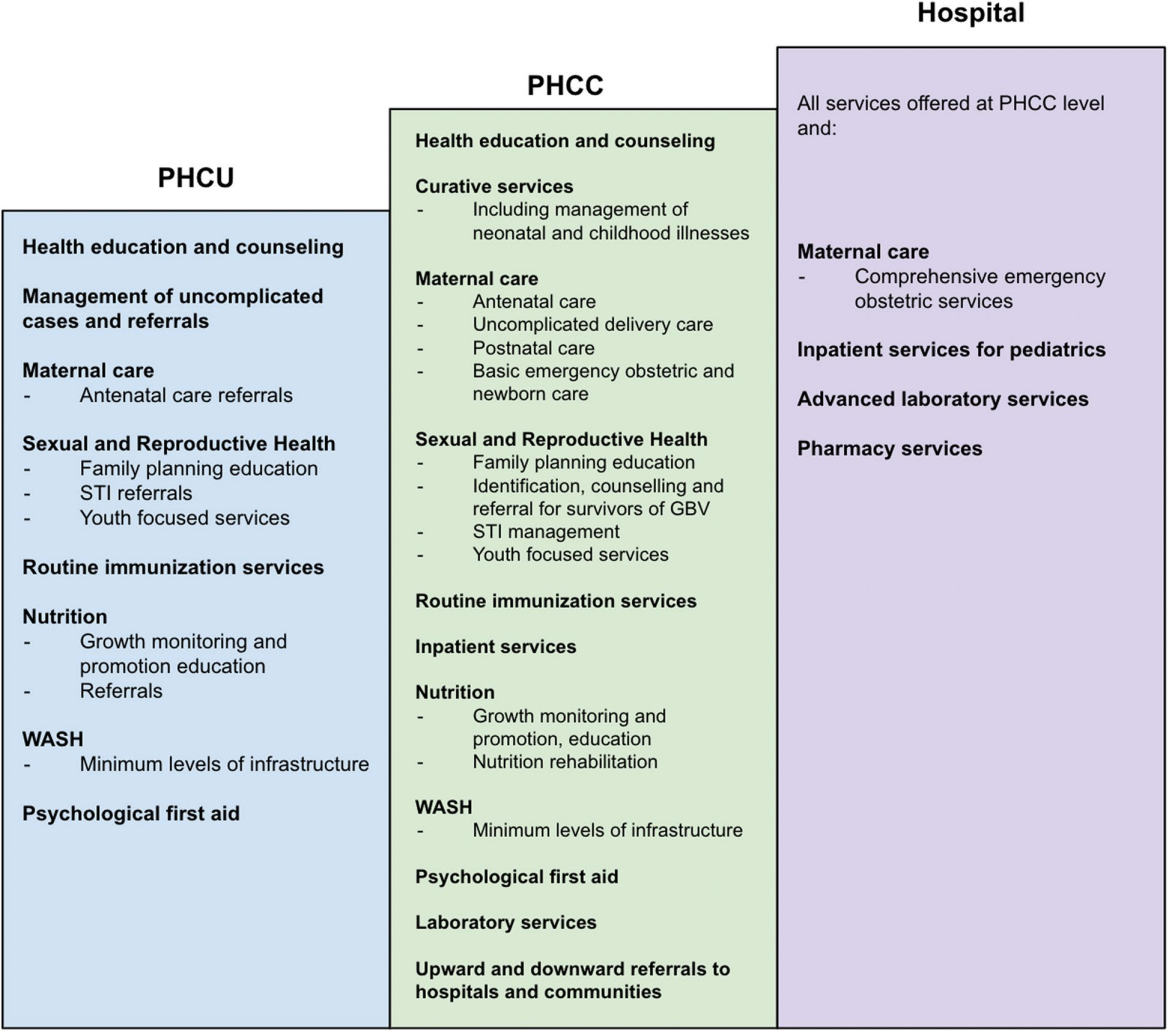
HPF service package by facility and service type

Figure 2 shows the overall prioritized service package for the HPF3, which IPs are contracted to implement (HPF Request for Proposals. Internally Circulated Document, Unpublished). These services are delivered through a network of community health workers and at primary health care units (PHCUs), PHCCs, and hospitals.

### Actors and their roles

**Fig. 3:**
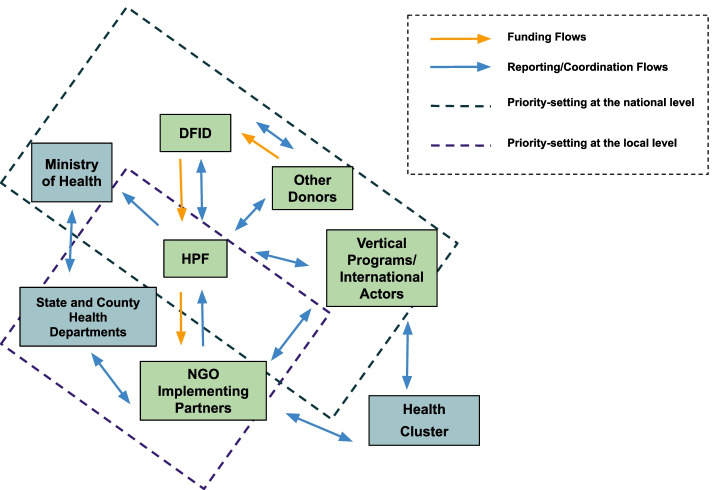
Overview of HPF actors and their relationships

Figure 3 shows the actors involved in HPF’s priority-setting and the funding, coordination and reporting flows among them. Funding streams from multiple donors, namely the United Kingdom's (UK) Department for International Development (DFID) (lead donor),^2^ Global Affairs Canada, the European Union (EU), the Swedish International Development and Cooperation Agency (Sida), the United States Agency for International Development (USAID), and Gavi, the Vaccine Alliance. DFID represents all donors and mainly sets the fund’s strategic direction. The MoH of South Sudan is the national health authority and a primary partner of HPF. It ensures HPF programming aligns with national health policy and strategic plans. The HFP fund is managed by a consortium of organizations, including Crown Agents (fund manager and IP contractor), International Procurement Agency (in-country warehousing and distribution), Montrose International (technical assistance in health service delivery, monitoring and evaluation, and communication), and the Royal Tropical Institute (operational research). The IPs are NGOs subcontractors engaged to provide and manage essential primary health care services through a network of health facilities and community based systems. IPs go through a competitive bidding process before being contracted. The SMoH is the link between the MoH and the CHDs and ensures that national policy is implemented at the local level. The CHD is the health authority at the county level. They work with the IP field teams to ensure facilities are operational and to collect information for the District Health Information System. Various international development partners (i.e. World Bank, UNICEF, Global Fund, and UNFPA) are also active in South Sudan implementing vertical and bilateral programs. Lastly, a health cluster aims to facilitate the coordination of funding flows within specific health areas to minimize the duplication.

### Formal and informal institutions

A formal institution related to HPF is the formal partnership between the MoH, donors, and HPF, with the aim of supporting the GRSS in delivering the BPHNS across eight states. The pooled funding is channeled directly to and managed by the fund manager, who maintains oversight over all financial resources and IPs, reports on progress to donors, and communicates directly with the MoH on operational issues (Fig. 3). The accountability mechanisms within these channels are shaped by formal contracts and arrangements between stakeholders to ensure the effective use of funds to achieve HPF’s key outcomes and deliverables.

Simultaneously, informal institutions are observed in the context of HPF. The formal institutional lines within the HPF structure are blurred because of MoH weaknesses in terms of coordination, technical capacity, and past misuse of funds. The donors provide substantial monetary and technical resources within the health sector via HPF and fill significant gaps regarding policy development and implementation. Although the donors and HPF work in collaboration with the MoH, the contracting mechanism, through an external fund manager, shifts a significant amount of power over the funds away from the MoH. Because funding remains outside the government system and national health budget, donors keep some decision-making agency, especially concerning fund management. According to a donor representative,

*We have a strong division between the meetings where we invite the ministry and the meetings with just the donors… We do communicate and engage them quite often...this program cannot be implemented [without]. But we still keep, especially the decision-making power with the donors. I can't tell you the amount of assets that have gone missing. So we have to be very careful with how we program and how much decision-making the ministry has...*

*This is the reason why it's a separate project and it's not going through the government, in any other context we would do budget support and we would engage with the MoH like that. –* Donor_02

Although the MoH is integral to HPF’s formal governing structure, the donors and HPF retain control over funding and its use. This is also displayed in their push for important policy decisions. For example, DFID was key in negotiating and moving forward the implementation of the national harmonized incentive scale.

*… [DFID] had a lot of the negotiations with the government and then [it was] finally published and adopted by the MoH... So that's one area that [DFID] pushed through and sort of used [its] leverage and the knowledge of the health sector in South Sudan to fight. –* Donor_01

Furthermore, the weak stewardship of the national MoH impairs communication channels with state- and county-level authorities. In the dearth of sustained consultation, the MoH does not adequately represent local authorities and their priorities and does not effectively relay national policy change and decisions. This amplifies the exclusion of local actors in national level decision-making.

### Interests and ideas among actors

HPF is made up of a complex web of actors at national and local levels with differing vested interests and motivated by particular ideas about health sector development. As lead donor, DFID is responsible for ensuring that HPF follows UK government commitments, which take a central role in HPF programming. A donor representative explained,

*[DFID is] responsible to ensure that HPF fulfills the mandate of the UK government that is expected of HPF... So HPF is run using DFID rules and regulations… [DFID sets the] strategic direction for the program and then the program implements that strategic direction, so that it’s in line with the UK government priorities of health… –* Donor_01

Donors must agree to adhere to DFID’s mandate and largely delegate their authority to DFID. However, individual donor interests do remain salient. Specific governmental/organizational requirements and mandates are considered in the creation of programming indicators. For example, donor interests to see improved tracking and results motivated the strengthened GESI component within HPF3’s service package. Overarching principles and ideals connected to larger donor government commitments to health drive these interests, in the case of GESI emphasizing social inclusivity and gender-sensitive rights.

These donor interests and ideas diverge from those of the MoH. From the MoH’s perspective, the HPF program is a government initiative implementing the national BPHNS. This is grounded in their mandate as the national health authority and their responsibility for the implementation of national health policies, i.e. National Health Policy, BPHNS, and the HSDP, to strengthen the health system. While the MoH is encouraged to take ownership over the health sector, HPF funding is tied to external accountability measures and interests, which complicates this ownership. According to an MoH official,

*There is nothing called HPF specific target or specific programming. There is one program and there is a government strategy in addressing the health issues and the key component we have now is the BPHNS and that includes minimum packages of health and nutrition...*—MOH_01

This misalignment was explained by an HPF team member,

*“[It] has been [a] misconception that the Ministry has, that...HPF money belongs to the people of South Sudan… Last year one of the big wigs at DFID wrote a letter and said... this money is donor money…it is not contributed by the MoH, so nobody can make demands and use it the way they want.” –* HPF_01.

Local level interests also diverge from those at the national level. The interests held by the CHDs are driven by their local needs, including strengthening local health infrastructure and governance through full coverage of facilities and support for renovations, infrastructure, training, and capacity building. Local authorities also expected HPF3 to continue in the same capacity as HPF2, with full coverage of facilities, payment of health workers’ salaries, and capacity building for the CHDs. According to an IP team member, CHD key priorities are,

*...related to capacity building, training… I think they are not so in line because most of the county authorities expected their routine of previous HPF to continue, for example, a lot of training...Also, most of the priorities of the county authority were focusing on renovations, building more facilities...more infrastructure, and then in HPF3 they are saying no, we don't have much support on building infrastructure... –* IP_08

Consequently, as the primary implementers of HPF programming at the local level, IPs need to negotiate these given their interest to adhere to the contractual obligations and priorities set by HPF3. Having specific contractual obligations on the one hand, and locally embedded knowledge and experience on the other, IPs need to balance these when making decisions about programming at the local level. In the experience of an IP team member,

*We follow the guidelines... because for us that is the donor and we have to follow their guidelines on what is their primary focus because they will come back and tell us this is what the donors want and so, we just have to adhere to them, even though you may have your own... experience... –* IP_05

### Priority-setting processes

#### National level

Priority-setting occurred at the national and local levels. National level priority-setting occurred in discussions determining the programming for HPF3, while local level priority-setting decisions were made by IPs throughout the bidding process and program implementation. At the national level, the process spanned several months prior to HPF3 from July to December 2018. A business case was developed by DFID and donors, which explicitly weighed and appraised three options for HPF3’s strategic direction. A *Community Health and Nutrition* focus was favored over a *Health Facility Infrastructure* and *National Health System Strengthening* focus based on certain criteria: cost-effectiveness (cost/disability adjusted life years (DALY) saved), coverage, equity, and health system stabilization. A community level focus was dominant in terms of cost-effectiveness (lowest costs/DALY) and had a positive assessment for coverage and equity. While this option was considered limited in terms of health system stabilization and strengthening, the GRSS was deemed unable to invest in health and nutrition beyond paying health workers’ salaries. As a result, the priority set forth by DFID was to orient funding toward the protection of life-saving health and nutrition services for the largest possible population in South Sudan (DFID HPF3 Business Case. Internally Circulated Document, Unpublished).

Priority-setting for the HPF programming service package occurred in a series of workshops (July-December 2018) led by DFID, including the Fund Manager, MoH and external actors including WHO, UNICEF and World Bank. Services from the BPHNS were selected to ensure HPF3’s objectives and outputs were met. Internal discussions occurred among the donors to align the package with their priorities. Broader consultative discussions also occurred with the MoH, the WHO, UNICEF, and the World Bank. In these discussions, the service package was reviewed against these partners’ activities to limit duplication. For example, funding for nutrition services was reduced because UNICEF is the key supplier of nutrition commodities and support for HIV services was minimized because the Global Fund provides HIV support through the UNDP. Lastly, the decision was made to reduce the number of facilities for HPF3, informed by an analysis of HPF2 facility data that showed no correlation between a high number of facilities and health outcomes. In general, it is not documented and thus unclear how *prioritized* services were discussed, weighed, and compared and whether this was completed in a systematic and rational way. Challenges were a lack of reliable data to inform decisions and the inapplicability of the BPHNS as a tool for priority-setting. The BPHNS was considered more as a ‘wish list,’ presenting a long list of interventions and without sufficient resources to be implemented. According to a donor,

*[Donor priorities] had to be mirrored with the BPHNS, which is quite broad and wide and sometimes a bit vague. So, a lot of that would fit within the BPHNS. –* Donor_01

### Local level

At the local level, priorities are set by IPs in the context of local interests and realities. National level decisions regarding HPF programming resulted in a RFP. This document provided potential IPs with an operational document to guide the bid preparation process. Under its guidance, potential IPs prepared a concept note and subsequent technical and budget proposals for a specific geographical area (lot). The proposal, budget preparation, and negotiation process was complex, requiring the management of technical requirements from HPF and (often divergent) local priorities while facing resource constraints and a dynamic environment.

Due to un-updated census numbers and inexact facility locations and information, IPs were required to bring specific local knowledge and experience to produce a competitive proposal. IPs were also expected to engage with CHDs to understand local priorities. It was frequently difficult to incorporate these priorities because they often diverged from HPF requirements, including the number of facilities to support. Some budget areas, including GESI, family planning, BHI and staffing were also mandatory, therefore necessitating the deprioritization of other areas to accommodate budget allocation to these key areas. This was explained by an IP team member.

*Because we were not able to cut from, say, family planning trainings, we couldn't cut from [GESI] trainings, those were mandatory. We couldn't cut any of the key identified staff... [and] BHI, because that had quite a prescribed set of activities… and we couldn't cut down on supervision either. So a lot of it came from the things that we had planned for facility management, [including] things like fixing up latrines, digging placenta pits, and doing small renovations. –* IP_11

IPs highlighted the absence of a “formula” and the complex and implicit nature of this process, as IPs negotiate and balance priorities until consensus is reached. This balancing act continued into the negotiation phase where the top-ranking NGO for each lot was invited for negotiations and given the chance to justify decisions in the proposal. This process took place between HPF, SMoH director generals, and the IPs. While this negotiation process was guided by explicit guidelines from the RFP, the resulting programming decisions were also dependent on the IPs’ ability to negotiate aspects of programming to maintain local buy-in. Although budget lines were relatively strict, some IPs explained that they could create some ‘decision space’ in their budget allocation. The number of facilities to be covered in a lot’s catchment area was a common point of discord because local authorities often contested the reduced number. In cases where controversy was high with the county-level officials, HPF agreed to raise the ceiling budget to accommodate more facilities.

*...the donors' pressures are very strong on our budget...we [were] asked to be allocating a certain amount on GESI...GAVI...BHI, so the budget was bound. Then at a certain point when I told them...I can do 1000 training on disabilities. Then the people with disabilities really have no facility to go to because they do not have enough funds to make facilities running... So, demonstrating that, quarreling a little bit, negotiating...I managed to distribute the funds as I wanted. –* IP_06

It is during the proposal development, review and negotiation process that local authorities (SmoH and CHD) were engaged in the priority-setting process. The impact of this engagement is observed in the disputes that occurred in certain IP proposal negotiations and indicates a level of engagement from certain local authorities. However, the extent to which this was consistent across states and counties is unclear. Full engagement is challenged by limited capacity, high turnover rates in SMoHs and CHDs, and limited channels of communication between national and state authorities with regards to policy changes and developments. Disagreements during proposal development, review, and negotiation from local authorities were often fueled by a lack of involvement, knowledge, and understanding of national level decisions with regards to national policy and the HPF program.

*... I had to allocate a certain percentage [to the BHI and] when I try to explain this to the SMoH... they told me, “You are crazy or what? You are cutting health facilities for Boma health workers?” and I said “This is the priority of your government and the donors, what should I do?” – IP_06*

**Fig. 4:**
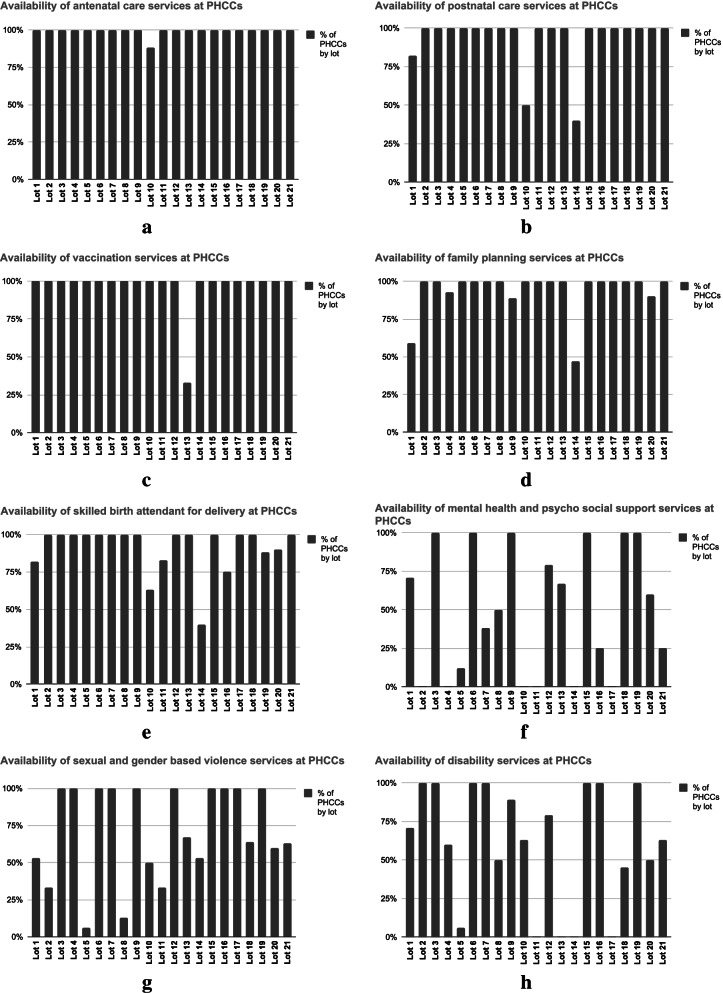
**a-h.** Availability of services at PHCCs across lots according to HPF’s 2019 Facility Service Register (HPF3 2019 Facility Service Register. Internally Circulated Dataset, Unpublished)

Implicit priority-setting decisions by IPs continue into implementation. Implicit decisions were made by IPs due to physical, cultural, monetary and supply-related constraints to service provision, leading to variation in service provision across lots. Figure 4 a-h shows the percentage of clinics per lot providing selected services from the HPF service package according to the HPF’s 2019 Facility Service Register. While little variation was observed for antenatal and immunization services, variation was found for family planning, skilled birth attendant, SGBV, MHPSS, and disability services. Key informants explained that this variation results from a lack of skilled health professionals. Access to health workers varies across the country, and it is especially difficult to secure an adequate workforce in areas where conflict is more concentrated. It is up to the IPs in collaboration with local field staff and local health authorities to mitigate these challenges. An IP team member explained,

*South Sudan.. overall, has a lot of challenges with qualified health personnel... A lot of the PHCUs [are] just run by community health workers. And in some situations, some of the PHCCs used to…and I'm sure some of them still are run by community health workers... So I think that poses a considerable challenge in trying to get the right level of service at some of these locations. – IP_11*

Service provision was also influenced by the IPs ability to mobilize resources to fill gaps in HPF funding. Health clusters are in place to track funding per technical area and facilitate this process. While IPs are encouraged to create synergies to access other funding sources and commodity streams, it is up to the IP to determine these gaps and access other sources of funding. This was described by HPF and IP team members,

*[We] encourage IPs to create synergies with other programs and donors. Especially for UNICEF and WHO and UNFPA...But in general we don't have expectations in terms of how they find those other HPF services. –* HPF_05

*It’s usually the initiative of the partners… We look at the data that comes out of the health facilities and we realize there is actually a need for the services in this area. And so based on that we usually can know who we can reach out to at the national level,..for instance, we've been having quite a long protracted discussion with the UNDP and the MoH to initiate ART services.—* IP_09

## Discussion

### Ownership and influence over the priority-setting process

This study has analyzed the political economy of priority-setting within the context of a multi-donor fund in a FCAS. One of the driving factors in the priority-setting process is the strong influence of external actors, in particular the donors through control of financial resources and technical expertise. The existence of power asymmetries between donors and government, leading to the former effectively setting the priorities for which health services to support is not unique to South Sudan and is commonly reported in other LMICs and FCAS [24, 25] largely driven by domestic pressures for donor agendas on international development, and a (not always openly expressed) concern for government corruption or limited bureaucratic capacity. Existing research on health financing reforms have stressed the importance of control over financial resources and technical expertise as critical mechanisms of influence on the priority-setting or policy making process, giving rise to sustainability concerns, inefficiencies and local ownership challenges [26–29].

These asymmetries are informed by and perpetuate the absence of government ownership throughout the priority-setting process. The contracting mechanism through which HPF is structured shifts a significant amount of decision-making authority away from the MoH and blurs the extent to which the MoH is involved in the priority-setting process. The discrepancy between the understanding of HPF’s position as a DFID led program following UK government commitments versus a GRSS initiative implementing national policy further amplifies this dynamic. This lack of ownership reflects a common concern in the use of contracting mechanisms in FCAS, over the extent to which they successfully promote government ownership and stewardship over the health sector [30, 31]. Afghanistan is an example where the use of a contracting mechanism has allowed for greater central responsibility over the management of health sector funding and services, with the crucial difference that external funding is channeled directly through the MoH, which contracts NGOs [32, 33]. Contrarily, the MoH in South Sudan is effectively bypassed through the HPF contracting mechanism and the institutional mechanism to integrate it into decision-making is weak.

These complex institutional mechanisms create an environment in which donor priorities largely guide programmatic decision-making. In the context of HPF several priorities were shared between the donors and the GRSS, namely the prioritization of maternal and child health services in HPF, which resembles the choices made in other low-income and crisis-affected settings, where these services have long been prioritized based on the argument that these provide good value for money [34–36]. Key shifts in the service package were driven by donor priorities. To start, the choice of a community health and nutrition focus was determined during a process internal to DFID. Furthermore, the increase in focus on GESI services was driven by a donor interest to strengthen the gender-sensitive dimensions of the HPF program and service package and made possible by an increase in targeted funding from the EU. The shift for donors to support a reduced number of facilities in order to prioritize the quality of services over the number of facilities supported by HPF was ultimately met with resistance by national and local health authorities. Furthermore, the change to an incentive payment system in HPF3 was facilitated by a national level policy decision to implement national harmonized incentive scales for the health workers, a decision that DFID was key in negotiating and for moving to implementation. These priorities do largely reflect domestic needs and global trends. For example, the focus on GESI ties to the emphasis that has been placed on Minimum Initial Service Packages for Sexual and Reproductive Health (SRH) in humanitarian health responses in recent years [37, 38]. Furthermore, the reduction of the number of facilities covered by HPF to focus on quality of care aligns with the increasing global attention to this topic in FCAS [39]. However, they are also driven by dominant technical, political, and economic donor interests. Documentation of these dynamics is important to further inform current debates in international development, reflecting broader asymmetry in the political economy of global health and international affairs [28].

The priority-setting landscape is further complicated by the insufficiency of the current BPHNS for South Sudan to provide guidance for priority-setting for the HPF service package. it is too expansive and unrealistic given financial and health system constraints. As a result, the BPHNS is used as a frame of reference for priority-setting but further processes determine the ultimate service package for HPF. This is a documented trend of basic packages of health services (BPHS) in many other contexts, where packages are too broad and are not linked with effective or clear benefits for population health [25, 40, 41]. Although such packages should be costed and realistic [28, 42], evidence suggests that BPHSs are routinely not connected to available resources or are unaffordable [4, 36, 40]. This leads to an “erosion of impact” as packages must undergo a second priority-setting cycle to reduce the set of services to be more in line with actual resources, [4, 40] and undermines the use of the BPHS as an explicit priority-setting tool. In practice, the selective support of specific services by health financing programs determines the extent to which communities access a range of primary health services [25]. In the context of HPF, the refinement of the package was led by donor priorities while considering the activities of other bilateral programs active in South Sudan. Although there were deliberative elements to the priority-setting process, through workshops and discussions involving stakeholders, the ways in which alternatives were valued, weighed, and discussed in the second priority-setting cycle are not clear and not documented. Such deliberative techniques have the advantage that they are able to address non-quantitative elements (especially in the absence of reliable data), explicitly enable the involvement of various stakeholders, and the ability to reach consensus [43]. Nonetheless, in the context of HPF the deliberative power was skewed in favor of the donors through the technical and financial resources they inherently bring to the priority-setting process, which puts into question whether consensus was meaningfully reached in the context of these power asymmetries.

These dynamics have also been highlighted by Khan et al. who in addition to the well-documented effects of direct and indirect financial and political incentives from donors and low investments into health on the part of governments on ownership over the policy process, reveal more subtle ways of control [28]. These include exclusionary practices when it comes to the utilization of knowledge for policy and planning. The inapplicability of the BPHNS as an explicit priority-setting tool eliminated an important point of entry and knowledge contribution into the priority-setting process for the MoH. The resulting process did not obviously ensure the inclusion of government stakeholders in priority-setting and planning.

### Local priority-setting dynamics

The study findings highlight the way priorities set at the central level are challenged and take shape when they come into contact with local interests and realities. IPs had to navigate the competing interests of the HPF program and local health authorities. The weak capacity of domestic health authorities at all levels and the dominance of external actors was found to limit the role of state and county health officials in this process, despite IP support to county health departments. Differences in priority-setting at the local level could be legitimate due to differences in health needs, the interests of local stakeholders and local realities [44]. However, the findings from this study suggest monetary constraints and contextual factors, including shortage of skilled health workers and conflict also influence priorities for service delivery. This leads to differences in the availability of services across the country and could result in inequities in healthcare access. Ultimately, the legitimacy of differences in service provision at local level, including SGBV services, remains difficult to assess without an explicit priority-setting process. This is exacerbated by the fact that priority-setting at the local level is extremely challenging due to the fact that contextual influences are greater, information is scarcer, and capacity is lower [45]. IPs had to make tough decisions when trying to balance programmatic priorities and local priorities. Similarly, studies in the Eastern Mediterranean region have shown that the process of contracting out services is a particularly political process that is affected by the wider policy context and could use guidance [46, 47]. Local priority-setting practices in the context of non-state contracting mechanisms and fragile localities warrant additional research to further understand and identify the modalities of decision-making at this level. This would also provide more insight into how these local processes can be guided while taking into account the political economy of local level decision-making with non-state and state actors.

### Use of PEA in low income fragile environments

Lastly, our study’s findings carry some implications for the future use of PEA as a tool in studying priority-setting or health financing policy and resource allocation processes more generally. In line with the argument made in relation to Zimbabwe [ 48], the findings from this study suggest PEA requires some adaptation in focus when applied to more low income, precarious and closed political environments. While party and pork barrel politics, as well as domestic patient, professional and industrial interest groups, i.e. interest group politics, play an important role in more middle or high income settings and competitive political environments [24], in the low income, conflict-affected and politically restricted setting of South Sudan these groups and mechanisms were found to play a less prominent role. Instead, external actor politics, with the involvement of a range of international development agents, were found to effectively dominate decision-making in this area. The contracting out model used, weak government capacity and the dominant role of donors and implementing International NGOs in this, also help to explain the limited influence of patronage politics concerns found in this study, as opposed to more stable and politically competitive elsewhere in Sub-Saharan Africa [49, 50]. The lack of evidence surfacing on this is however also a result of the limitation of the study scope.

### Study strengths and limitations

To our knowledge this is the first study that uses an adapted political economy policy analysis framework to assess priority-setting in a FCAS and in the context of a multi-donor fund. As opposed to an evaluation against a more universalist ethical framework, this study has provided insights in the interactions between the context, actors and processes that shape the environment in which priorities are set. Nevertheless, this study has been subject to several limitations. Firstly, information bias could have occurred due to a lack of formal documentation on the priority-setting processes occurring at the national and local level. However, during the key informants interviews the most essential elements were discussed. Secondly, selection bias could have influenced our findings, as local health authorities (SMoH and CHDs) were less represented and citizen representatives were not consulted. Nonetheless, steps were taken to ensure representationfrom the main stakeholder groups, including local authorities. This representation by local authorities remained more limited, however, due to technical, logistical, and language barriers. Thirdly, recall bias may have influenced re-call of the priority-setting processes that took place over a year ago as respondents may have forgotten key details. Fourthly, the facility register used for the quantitative data analysis on the provision of services included self-reported data. While it may have led to a different picture in terms of variation across lots, the overall conclusion that variation exists likely still holds. Furthermore, it is important to note that these results are time-bound to the period of research and could not take into account the Covid-19 pandemic that began during data collection and undoubtedly affected the priority-setting landscape of HPF3 post-2020. However, the dynamics documented in this study are unlikely to have changed fundamentally, and its implications remain relevant to consider for priority-setting for health in comparable low resource, FCAS. Lastly, some strengths and limitations pertain to the positionality of the research team members. The positionality of the lead author may have had both a beneficial and constraining effect on the research process. Although associated with HPF, the independent role and outsider status of the lead author might have helped to lessen the power asymmetry in the relationship with interviewees outside of HPF [51]. However, this detachment might have also been a limitation in terms of a feeling of unfamiliarity on behalf of the interviewees, who might have deemed the researcher to be less able to appropriately contextualize the findings. This was mitigated by the active participation of research team members who were closer to ‘insider status’ on the insider–outsider continuum in the research design and data analysis phases [52].

## Conclusion

This study shows that pervasive power asymmetries between donors and national health authorities exist in the context of priority-setting for the HPF in South Sudan. The process of contracting out services to NGOs is highly political and the priority-setting process seems to be dominated by donor agendas, leaving little room for government ownership and balanced participation. Overall, the HPF program priorities are in line with global trends, with emphasis on value for money, MNCH, GESI, and community health structures. While the priority-setting process for the program development was deliberative with involvement of stakeholders, it is not documented how interventions were valued, weighed, and discussed. At the local level, priorities were further refined by NGOs that had to balance HPF program needs with local health authorities' interests. Furthermore, local realities such as conflict and health systems constraints ultimately determined the service package for and coverage by HPF. The BPHNS of South Sudan was found to be overly ambitious given financial and health system constraints, and therefore provides insufficient guidance for priority-setting. With a focus on the interplay between the low resource, conflict-affected setting and stakeholders’ interests and its effect on the use of scarce resources, this study has shown the importance of considering the political economy of health priority-setting in FCAS.

## Data Availability

The datasets generated and/or analysed during the current study are not publicly available due to protection of participants (given the difficulty of fully anonymising qualitative transcripts) and because the quantitative data which informed analysis is monitoring data that has not been publicly circulated.

## Abbreviations

BHI: Boma Health Initiative
BPHS: Basic Package of Health Services
BPHNS: Basic Package of Health and Nutrition Services
CHD: County Health Department
DALY: Disability Adjusted Life Years
DFID: Department for International Development
EU: European Union
FCAS: Fragile and Conflict Affected Settings
GESI: Gender Equality and Social Inclusion
GRSS: Government of the Republic of South Sudan
HPF: Health Pooled Fund
HSDP: Health Sector Development Plan
Ips: Implementing Partners
LMIC: Low- and Middle-Income Countries
MHPSS: Mental Health and Psychosocial Support
MoH: Ministry of Health
NGOs: Non-governmental organizations
PEA: Political Economy Analysis
PHCC: Primary Health Care Center
PHCU: Primary Health Care Unit
RFP: Request for Proposals
SGBV: Sexual and Gender Based Violence
SmoH: State Ministry of Health
SRH: Sexual and Reproductive Health
UK: United Kingdom

## Footnotes

1 Central Equatoria, Eastern Equatoria, Lakes, Northern Bahr el Ghazal, Unity, Warrap, Western Bahr el Ghazal and Western Equatoria.

2 DFID was replaced by the Foreign, Commonwealth & Development Office in September 2020, just after data collection. As this study is retrospective, this paper will continue to refer to FCDO as DFID.
